# International Biological Engagement Programs Facilitate Newcastle Disease Epidemiological Studies

**DOI:** 10.3389/fpubh.2015.00235

**Published:** 2015-10-19

**Authors:** Patti J. Miller, Kiril M. Dimitrov, Dawn Williams-Coplin, Melanie P. Peterson, Mary J. Pantin-Jackwood, David E. Swayne, David L. Suarez, Claudio L. Afonso

**Affiliations:** ^1^Exotic and Emerging Avian Viral Diseases, Southeast Poultry Research Center, United States Department of Agriculture – Agricultural Research Service, Athens, GA, USA; ^2^National Diagnostic and Research Veterinary Medicine Institute, Sofia, Bulgaria; ^3^Office of International Research Programs, George Washington Carver Center, United States Department of Agriculture – Agricultural Research Service, Beltsville, MD, USA

**Keywords:** newcastle disease, NDV, APMV-1, surveillance, biological engagement programs, wild birds, poultry

## Abstract

Infections of poultry species with virulent strains of Newcastle disease virus (NDV) cause Newcastle disease (ND), one of the most economically significant and devastating diseases for poultry producers worldwide. Biological engagement programs between the Southeast Poultry Research Laboratory (SEPRL) of the United States Department of Agriculture and laboratories from Russia, Pakistan, Ukraine, Kazakhstan, and Indonesia collectively have produced a better understanding of the genetic diversity and evolution of the viruses responsible for ND, which is crucial for the control of the disease. The data from Kazakhstan, Russia, and Ukraine identified possible migratory routes for birds that may carry both virulent NDV (vNDV) and NDV of low virulence into Europe. In addition, related NDV strains were isolated from wild birds in Ukraine and Nigeria, and from birds in continental USA, Alaska, Russia, and Japan, identifying wild birds as a possible mechanism of intercontinental spread of NDV of low virulence. More recently, the detection of new sub-genotypes of vNDV suggests that a new, fifth, panzootic of ND has already originated in Southeast Asia, extended to the Middle East, and is now entering into Eastern Europe. Despite expected challenges when multiple independent laboratories interact, many scientists from the collaborating countries have successfully been trained by SEPRL on molecular diagnostics, best laboratory practices, and critical biosecurity protocols, providing our partners the capacity to further train other employes and to identify locally the viruses that cause this OIE listed disease. These and other collaborations with partners in Mexico, Bulgaria, Israel, and Tanzania have allowed SEPRL scientists to engage in field studies, to elucidate more aspects of ND epidemiology in endemic countries, and to understand the challenges that the scientists and field veterinarians in these countries face on a daily basis. Finally, new viral characterization tools have been developed and are now available to the scientific community.

## Introduction

Newcastle disease (ND) is one of the most significant diseases of poultry worldwide. It is caused by virulent strains of Newcastle disease virus (NDV), also known as avian paramyxoviruses of serotype 1 (APMV-1). The presence of virulent viruses in poultry must be immediately reported to the World Organisation for Animal Health (1). From 2012 through 2014, infection with virulent NDV (vNDV) was suspected or reported to the OIE by 58 countries as being present in domestic poultry, with an additional 5 countries reporting the disease in limited zones.^1^ During this same time period, 15 countries reported vNDV in wild birds to the OIE. Many countries do not adhere to OIE reporting guidelines for vNDV, as the disease is endemic; therefore, the presence of the virus is underreported. The true prevalence of vNDV in the wild-bird population is not reflected in the limited number of reports, since wild-bird species are not routinely studied. Except for studies on migratory birds (which are principally surveyed for the presence of avian influenza) there are few available funds for the surveillance of diseases in other wild-bird species, and logistically sampling wild birds is difficult due to the regulations protecting their capture.

The greatest impact of ND is due to the high mortality rates on village poultry, reducing egg, and meat protein availability. Village or smallholder poultry production is a major source of protein and income for small farmers, and outbreaks of ND contribute to food insecurity in these communities. In addition, ND also affects intensive production facilities worldwide by triggering the implementation of economically significant trade restrictions, and by increasing costs of production from culling and quarantines for the infected premises. vNDV is also considered as a select agent and a potential bioterrorism threat agent in the United States (US). Whether in domestic poultry or wild birds, vNDV strains remain a threat to all producers of poultry.

## Biological Engagement Programs

In 1998, the US Department of State (DOS) assisted selected former Soviet chemical and biological weapons scientists to redirect their efforts to peaceful, agricultural research and to help reduce the proliferation of weapons of mass destruction. Almost a decade later, the DOS Biosecurity Engagement Program (BEP) was implemented with the broader mission of providing financial and intellectual assistance to microbiological laboratories to enhance biosecurity, biosafety, and pathogen and disease surveillance, while decreasing biological threats, globally. The collaborations expanded past the former Soviet Union (FSU) to include South and Southeast Asia, the Middle East and Africa. One area of common mutual interest across these regions was scientific collaboration on avian influenza virus (AIV) and NDV, both notifiable and listed diseases to the OIE.

In 2000, a long-standing collaboration between the ARS Southeast Poultry Research Laboratory (SEPRL) and Russian counterparts began on avian influenza and NDV and provided unique opportunities for surveillance and research in the region. In 2002, SEPRL began collaborating with partners in Kazakhstan; in 2010, collaborations began with Ukraine, Egypt and Indonesia; and in 2011, with Pakistan. From 2000 to 2015, SEPRL has collaborated on 14 bio-engagement research projects on avian influenza and ND with its global partners mainly funded by the DOS, but also more recently by the Department of Defense – Defense Threat Reduction Agency. While most of the initial interactions with foreign laboratories often began with the training of collaborators at SEPRL, formal scientific collaborations often developed subsequently (Table 1).

**Table 1:** **Contact information and country of origin for all collaborating institutions**.

Institution contact information	Country
National Diagnostic and Research Veterinary Medicine Institute, 15 Slaveikov Blvd, Sofia 1606, Bulgaria	Bulgaria^a^
Laboratory of the Ministry of Agriculture, Tbilisi Laboratory of the Ministry of Agriculture (2), 65 Godziashvili str, Tbilisi 0159, Georgia	Georgia
Faculty of Veterinary Medicine, Bogor Agricultural University, Jl. Agatis Kampus IPB Dramaga, Bogor 16880, West Java, Indonesia	Indonesia
Gadjah Mada University, Faculty of Veterinary Medicine, Jl. Fauna 2, Karang Malang, Yogyakarta 55281, Indonesia	Indonesia
Kimron Veterinary Institute, Israel, Division of Avian Diseases, Bet Dagan, P. O. Box 12, Israel 50250	Israel^a^
Institute of Microbiology and Virology, 103, Bogenbai batyr Str., 480100, Almaty, Kazakhstan	Kazakhstan
Research Institute of the Biosafety Problems (RIBSP) 19–13, Gvardeisky, Kordaiskiy 080409, Kazakhstan	Kazakhstan
Comisión México Estados Unidos para la prevención de Fiebre Aftosa, Senasica Rio Pánuco #852 Fracc, Los Laguitos, Edificio SAGARPA C. P. 29029 Tuxtla Gutiérrez, Chiapas Mexico	Mexico
National Veterinary Research Institute, PMB 01 Vom, Plateau State, Nigeria	Nigeria^a^
Hivet Animal Health Business 667-P, Johar Town, Lahore, Pakistan, 45000	Pakistan
Quality Operations Laboratory/Institute of Biochemistry & Biotechnology, University Of Veterinary and Animal Sciences, Lahore, Pakistan 45000	Pakistan
D.I. Ivanovsky Virology Institute, Minzdravsocrazvitia Rossii, Scientific Production Association “Narvac” 16 Gamaleyi St., Moscow, Russia, 123098	Russia
“Federal Centre for Animal Health” (FGI “ARRIAH”). 600901 Yur’evets, Vladimir, Russia	Russia
Novosibirsk State University, Department of Research, Novosibirsk, Russia 630090, (383)–330–3244, and Division of Emerging Zoonotic Diseases and Influenza. State Research Center of Virology and Biotechnology “Vector,” 630559, Koltsovo, Novosibirsk region, Russia	Russia
Department of Microbiology and Biotechnology, School of Biological Sciences, University of Dodoma, P. O. Box 259, Dodoma, Tanzania	Tanzania
Department of Veterinary Medicine and Public Health, Sokoine University of Agriculture, P. O. Box 3021, Chuo Kikuu, Morogoro, Tanzania	Tanzania
National Scientific Centre, “Institute of Experimental and Clinical Veterinary Medicine,” 83, Pushkinskaya Street, Kharkiv 61023, Ukraine	Ukraine
Emerging and Exotic Avian Viral Disease Research Unit, Southeast Poultry Research Laboratory, US National Poultry Research Center, USDA/ARS, 934 College Station Road, Athens, GA 30605, USA	USA

*^a^Collaboration not funded by BEP*.

The primary role of SEPRL in these collaborations was to analyze, coordinate, integrate, centralize, and share epidemiological information among partners. Another key role was to develop and provide standard protocols and operating procedures to allow data from multiple institutions to be compared. The collaborating institutions provided expertise for each of their localities concerning management practices necessary for field surveillance and sample collections.

### Training

The training objectives and goals for foreign collaborating scientists have been diverse and adapted to their respective needs, depending on their country of origin. Representatives of Afghanistan, Azerbaijan, Brazil, Egypt, Georgia, Indonesia, Kazakhstan, Kenya, Libya, Malaysia, Mongolia, Mexico, Morocco, Nigeria, Pakistan, Palestine, Russia, and Yemen have received different levels of training at SEPRL. The BEP collaborations began by building on the success that SEPRL previously established through international collaborations with the International Science and Technology Center (ISTC), Food and Agricultural Organization (3), US Agency for International Development (USAID), Foreign Agricultural Service (4), Animal and Plant Health Inspection Service (5), and Wildlife Conservation Society funded projects collaborating with Japan, South Korea, Indonesia, Vietnam, Russia and FSU countries, e.g., Kazakhstan. These collaborations have built a network of avian influenza and ND scientists around the world, which have yielded joint research projects, published scientific studies in peer-reviewed journals, resolved trade issues on poultry products, and improved competence of all of the scientists involved. Objectives included in all collaborative training modules have improved diagnostic assays and enhancement of biosecurity. For diagnostics, visiting scientists have learned techniques of egg inoculation for virus isolation and identification, real-time reverse-transcriptase PCR (rRT-PCR) for the detection of NDV, and methods of differentiation of vNDV from NDV of low virulence utilizing rRT-PCR. Techniques and basic principles of primer designs, sequencing, sample preparation, phylogenetic tree construction, and serology (hemagglutination-inhibition [HI] assay and ELISA) were transferred during the collaboration. Biosecurity and containment operations training included laboratory safety, proper safety data records, handling compressed gas cylinders, Occupational Safety Health Administration (OSHA) laboratory standards, general biosecurity, biohazard risk assessment and management, disinfection and sterilization, personal protective equipment, emergency and spill response, engineering controls, and shipping regulated biological materials. For a selected group of scientists that received extended training, other modules offered included working with the Institutional Animal Care and Use Committee (IACUC), which allowed them to be included on animal use protocols and to be involved with animal studies.

During the research projects, SEPRL scientists visited their foreign counterparts in Russia, Kazakhstan, and Ukraine 32 times. SEPRL scientists also hosted 30 collaborators for scientific visits involving research and training. Under the auspices of the projects, SEPRL and foreign collaborators participated in over 25 international conferences in the US, Russia, Ukraine, South Africa, Spain, Germany, China, Latvia, the United Kingdom, and Thailand. In addition to the research projects, from 2008 to 2010, SEPRL provided training on molecular techniques to detect AIV and NDV to 57 scientists from 21 countries. Additional informal collaborations with scientists from Israel, Bulgaria, Nigeria, Tanzania, and Mexico have added to the body of knowledge on AIV and NDV epidemiology. Through training and collaborative research efforts, SEPRL has developed an extensive network of scientists around the world working on AIV and NDV.

The initial objective of the scientific proposals between SEPRL and the collaborating laboratories included increasing the number of surveillance samples obtained from commercial and domestic poultry species, and also wild birds. Scientists from the participant laboratories processed these samples together with SEPRL scientists, identified and characterized the strains of NDV obtained. Subsequent testing of molecular diagnostic techniques documented if routinely used molecular assays were able to detect all of the viruses isolated. Additional characterization of isolates, such as pathogenesis studies in chickens, vaccine studies, or phylogenetic sequence analysis was performed depending on the scientific interests or industry concerns. The objective of the training was to prepare foreign scientists to be able to accurately and promptly detect vNDV and to identify what avian species the viruses are likely to be obtained from. Those skills were considered to be critical for preventing, containing, and controlling ND outbreaks. Furthermore, efforts were made in promoting safe, secure, and responsible use of biological materials that are at risk of accidental release or intentional misuse as the prevention of accidents improves the quality of life for people from all the countries involved.

### Molecular Epidemiology of Lentogenic Viruses

NDV of low virulence, also known as lentogenic NDV, are widely distributed worldwide and are present in multiple avian species that may be in contact with poultry. Lentogenic viruses are used as vaccines to prevent mortality and clinical disease from vNDV infections in poultry. However, some lentogenic strains produce respiratory infections that decrease productivity in poultry, and have the potential to become virulent upon mutation of the cleavage site of the fusion protein gene. The nucleotide sequence of the fusion gene is used to classify NDV strains into genotypes and the cleavage site sequence of the fusion protein of NDV is the accepted OIE identifier of virulence. Strains that have three or more basic amino acids at positions 113–116 with a phenylalanine at position 117 are by definition virulent (1). Since scientists began to analyze NDV strains phylogenetically, the fusion gene nucleotide sequence has been and continues to be the standard gene used to classify NDV strains and to study virus evolution (6, 7). Although infection with vNDV is one of the greatest concerns for poultry producers, a better understanding of the epidemiology of lentogenic NDV is also very important, as transmission of these viruses from poultry to wild birds, and vice versa, has been previously documented. The distribution of lentogenic NDV in wild birds has been studied by our group and by others, however, the full extent of the interactions between lentogenic NDV from wild birds and poultry is largely unknown (8–10). As most lentogenic viruses are normally detected using the same diagnostic assays (serology and real-time PCR) as virulent viruses, the interpretation of some previously published epidemiological data, that does not involve sequencing or that distinguish vNDV from NDV, is difficult. Thus, efforts have been made to train and to develop sequencing capacities in partner countries.

A collaboration project with the National Scientific Center Institute of Experimental and Clinical Veterinary Medicine, Kharkiv, Ukraine was initiated to study the geographic distribution, wild host species distribution, and ecological factors affecting virus transmission of avian paramyxoviruses in Ukraine. Wild-bird surveillance was conducted in Crimea, Kherson, Zaporizhia and the Donetsk regions of Ukraine. The Black and Azov Sea regions are part of an intercontinental (north to south and east to west) flyway, and the similarity of the NDV strains isolated from these regions with other NDV from Northern Europe and Africa confirmed the importance of sampling birds in these regions for the early detection of viruses transported by wild birds (11).

Surveillance for hemagglutinating (HA) avian paramyxoviruses was conducted during 2006–2011 through different seasons of the year on 6,735 wild birds, representing 86 species, from 8 different orders. The presence of HA positive viruses in oral and cloacal swabs was obtained and the serotypes of the APMV were identified by hemagglutination-inhibition tests. The APMV obtained from swabs were serologically characterized and determined to belong to different serotypes as follows: APMV-1 (*n* = 9), APMV-4 (*n* = 4), APMV-6 (*n* = 3), and APMV-7 (*n* = 4) (11). Overall the highest viral isolation rate occurred during north to south autumn migration with viruses isolated mainly from mallards, teals, dunlins, and a widgeons at rates ranging from 1.9 to 25% depending on species and location. The rate of isolation was lower during winter (December–March) (0.32%), with viruses isolated mainly in the Black and Azov Sea regions from ruddy shelducks, mallards, white-fronted geese, and a starling (12). Surprisingly, during spring migration, and the time that included nesting (April–August), no APMV strains were isolated out of 1,984 samples tested. Sequencing and phylogenetic analysis of four APMV-1 and two APMV-4 viruses showed that one APMV-1 virus belonging to class I was epidemiologically linked to viruses from China, three class II APMV-1 viruses were epidemiologically connected with viruses from Nigeria and Luxembourg, and one APMV-4 virus was related to goose viruses from Egypt. In summary, multiple wild-bird species likely to be infected with different types of APMVs were identified, and the data supported the existence of a possible Africa-Europe route of intercontinental transmission of APMVs by wild birds.

As a result of an international collaboration that included the US Geological Survey at the Alaska Science Center in Anchorage, the Southeastern Cooperative Wildlife Disease Study group of The University of Georgia, Athens, GA, USA, the Research Center for Animal Hygiene and Food Safety, Obihiro University of Agriculture and Veterinary Medicine in Inada, Hokkaido, Japan, and the State Research Center of Virology and Biotechnology ‘VECTOR’, Novosibirsk Region, Koltsovo, Russia, the genetic diversity of APMV-1 isolated from migratory birds connected with Alaska and the continental US was studied (13). Swabs samples from migratory birds from the US, Japan and Russia were assessed for the evidence for north-south and east-west intercontinental virus spread. Viruses were isolated and sequenced, phylogenetic methods of tree construction based on maximum likelihood and prediction of viral virulence by sequence analysis were utilized to compare isolates of APMV-1 strains isolated from migratory birds from Alaska, Japan, and Russia. A total of 73 APMV-1 isolates were sequenced as part of this study. Most isolates were closely related to lentogenic viruses from class I and II genotypes previously reported to be present in wild birds (8).

Analysis of the fusion protein sequence data revealed that none of the putative amino acid sequences for the fusion protein cleavage sites were consistent with those of previously identified virulent viruses. Besides the fact that five isolates of genotype I of class II formed a monophyletic cluster exhibiting previously unreported genetic diversity, which met criteria for the designation of a new sub-genotype, the most significant finding of this study was the close genetic relationship among selected isolates from wild-bird isolates from widely divergent geographic location. Close relationships were found among viruses of class II, with Russian NDV strains closely related to North American viruses (class II sub-genotypes Ib and Ic). More specifically NDV strains from Alaska (US) and Japan were related to NDV from Maryland (US), suggesting multiple opportunities where birds from different areas were exposed to each other’s viruses. The relatedness of these viruses provided indirect evidence for possible intercontinental virus spread by migratory birds. Furthermore, the close relationship between NDV from class II genotype I, sub-genotypes Ib and Ic, confirmed that migratory bird movement is one of the possible mechanism for the redistribution of NDV strains (13). Due to the location and the intensity of the surveillance, most class I viruses were from North America. Interestingly, two isolates, one each from Japan and Russia were closely related to American viruses of class I, sub-genotype 1c. These data, however, did not provide support for the hypothesis that wild-bird strains may be contributing to the emergence of more vNDV as, none of the isolates resembled known virulent viruses. The estimated mutation rates for fusion genes of class I and class II wild-bird strains were within the values typical for NDV strains and there was no evidence of vaccine viruses present in waterfowl. Thus, these studies provided new insights into the diversity, spread, and evolution of lentogenic NDV in migratory wild birds, worldwide.

### Epidemiology of vNDV Strains that Cause ND

The epidemiological factors responsible for the maintenance and spread of vNDV strains are largely unknown. It is suspected that vNDV may persist in vaccinated animals, however, repeated isolations of vNDV strains in wild birds also suggests the existence of natural reservoirs in birds species that either might not be susceptible to disease or may be partially resistant, such as parrots, cormorants and pigeons. Evaluating surveillance samples of domestic poultry species along with samples from wild-bird populations geographically surrounding them is critical for completely understanding the epidemiology of ND.

The BEP collaborations between SEPRL and Pakistan involved two departments of the University of Veterinary and Animal Sciences, in Lahore; (1) Quality Operations Laboratory and (2) the Pakistan Institute of Biochemistry and Biotechnology, and another institution, the Poultry Disease Diagnostic Laboratory in Gakkhar, Gujranwala, Punjab. Efforts were focused on epidemiological studies designed to identify and characterize circulating strains of NDV in Pakistan. The first BEP collaboration with Pakistan characterized eight vNDV strains isolated from the years 1974, 1995, and 2004 through 2008 (14). Besides characterizing by sequencing a 374 base pair (15) region of the fusion gene that is critical to determine virulence, the mean death time (MDT) and intracerebral pathogenicity index assay (ICPI) were performed to confirm their virulence. The isolates from 2007 and 2008 grouped into distinct cluster within genotype VII and were most closely related to a vNDV isolated from Japan in 1989. These viruses later proved to be an entirely distinct genotype XIII (7), later noted as XIIIb (16). The original NDV strains of genotype XIII (now renamed sub-genotype XIIIa) from South Africa, Iran and India may have given rise to an additional sub-genotype (XIIIb) now present in Pakistan. Intracerebral pathogenicity and assessment of gross and histological assessment of representative strains for sub-genotype VIIi and genotype XIIIb show intracerebral pathogenicity indices (ICPI) of 1.89 and 1.85, and 100% mortality within 4 and 5 days, respectively (17). The ICPI scale goes from 0 to 2, with a score close to zero meaning no morbidity or mortality, a score equal to or greater than 0.7 meaning a virulent and reportable virus, and a score closer to 2 meaning a highly virulent virus in poultry (1). Therefore, the Pakistani viruses were highly virulent.

The majority of the remaining Pakistani strains grouped with variants of NDV found in pigeons from genotype VIb, often called pigeon paramyxovirus-1 (PPMV-1). PPMV-1 are virulent by definition since they contain three or more basic amino acids at positions 113–116 and a phenylalanine at position 117 of the fusion protein cleavage site and have an ICPI of equal to or greater than 0.7, thus are reportable to OIE (1). While these isolates are found worldwide in pigeons, they show varying rates of clinical disease upon infecting chickens. The Pakistani collaborations have continued and remain on going. As a result, besides genotypes XIIIb and VIb two other NDV genotypes were identified from circulating vNDV at different times in Pakistan and the surrounding countries. These included vNDV strains of the most widely distributed genotype VIId, and a newer sub-genotype VIIi that have been found in Pakistan and the Middle East.

Most recently, our international collaborations have broadened to include collecting data to improve the current knowledge concerning the mobility and evolution of NDV genotypes circulating since 2011. These studies incorporated collaborations with the Kimron Veterinary Institute, from Bet Dagan Israel, and the Department of Infectious Diseases and Veterinary Public Health, of the Veterinary Medicine-Bogor Agricultural University, in Bogor, Indonesia. These studies lead to the discovery that vNDV had rapidly spread across continents into three countries that are not geographically connected (16). Viruses of sub-genotype VIIi were found to be rapidly spreading through Asia and the Middle East causing outbreaks of ND in vaccinated birds, wild birds, and pets (18). A close phylogenetic relationship with viruses previously isolated in wild birds in Indonesia was found and it was suggested that permanent uncharacterized reservoirs of NDV might exist in wild birds. The discovery of viruses of sub-genotype VIIi in vaccinated animals (19) suggested that this new sub-genotype has the potential to be maintained in vaccinated animals. Additionally, the presence of significant disease on farms since 2011 until 2013 throughout Indonesia, Pakistan, and Israel caused by viruses of this sub-genotype highlight its potential to cause a new panzootic (16). Further evidence supporting these findings is the introduction of this virus into Eastern Europe (16).

Another new sub-genotype, VIIh, has also been identified in Indonesia, Bali, China, and Malaysia from viruses isolated from 2007 through 2011. Most significant is the fact that the viruses of new genotypes seem to be replacing older viruses. In Pakistan, the viruses of sub-genotype VIIi have replaced NDV isolates of genotype XIII, which were commonly isolated in 2009–2011, and they have become the predominant sub-genotype causing ND outbreaks since 2012. Similarly viruses of sub-genotype VIIi were isolated in Israel in 2012, while the presence of the previously predominant sub-genotypes VIId and VIIb, decreased during that year.

Although the viruses of one of the most commonly isolated sub-genotypes of genotype VII (VIId) has been gradually replaced by new viruses in some countries of Asia and the Middle East, these older viruses of VIId have continued to circulate worldwide. Three viruses isolated from chickens in Ukraine between 2003 and 2013 have been classified as members of sub-genotype VIId. Interestingly, these viruses resembled NDV isolates from backyard poultry in Bulgaria from 2006 and 2007 (unpublished data). These viruses were closely related to isolates from China from 2003 to 2007 and Serbia from 2007, further providing evidence for the distribution patterns of vNDV.

The earliest reports of ND outbreaks in Kazakhstan were reported in 1980 and despite regular vaccination of domestic poultry the disease is thought to be endemic in the country (2). Twenty-eight vNDV strains from chickens from Kazakhstan and Kyrgyzstan isolated from 1998 through 2005 were found to have virulent fusion protein cleavage sites (^113^-R-Q-R/K-R-F-^117^) and ICPI values ranging from 1.05 to 1.87 (20). Similar to what happened in Pakistan; the strains isolated from chickens in 1998 through 2001 of sub-genotype VIIb were replaced by strains from sub-genotype VIId from 2003 onward. Altogether, during this collaboration 38 chicken strains were sequenced and characterized. Additional work on three variant NDV strains (PPMV-1) from a 2005 ND outbreak in pigeons found these strains to be similar to other classical PPMV-1 of sub-genotype VIb clustering with other mesogenic (moderately virulent) viruses from Poland, Austria, and Croatia (21).

Similarly, our Russian collaborators evaluated 77 strains from sick or dead domestic and feral pigeons collected from 2001 through 2009 from 17 administrative divisions. While seven of these strains grouped with the same sub-genotype, VIb as the strains from Kazakhstan, 70 of the strains grouped into a different sub-genotype of VIb with 52 located in the European portion of the country and 18 in the more easterly Siberian portion (22). With ICPI values ranging from 0.80 to 1.4, these strains were also considered to be mesogenic. The presence of one of the Siberian strains from a bird in the European portion of the country demonstrates that viruses of pigeons do move across large distances regardless of geographical borders.

USDA offshore funding to study the epidemiology of vNDV in Mexico allowed the identification of vNDV from a sub-genotype Vb, circulating in poultry. Furthermore, the isolation of NDV of low virulence, commonly used as live NDV vaccines in several species of wild birds, provided evidence to suggest the existence of epidemiological links and more than one spillover from poultry into the environment (10). Additional characterization of circulating viruses in Mexico and Central America allowed designation of a new sub-genotype, namely Vc. Three Belize isolates from 2008 were nearly identical to a Honduras isolate from 2007 (sub-genotype Vb), but distinct from viruses circulating in Mexico (also sub-genotype Vb) during the same time period, thus suggesting separate evolution among viruses from Mexico and Central America (23).

An additional collaboration with Tanzania analyzed surveillance samples from live bird markets in five different areas of the country with vNDV isolated from 10% of the samples and further characterization of these samples is ongoing (unpublished data).

Thus, the evidence suggests that the simultaneous evolution of vNDV strains from multiple genotypes has continued in different locations and different hosts since the first vNDV were identified in 1926. However, increased surveillance, and prompt epidemiological characterization of new strains circulating in developing countries funded by BEP and other international efforts have allowed a better understanding of the evolution of vNDV and has increased awareness that ND is a global problem.

### Diagnostics

One of the goals of the BEP collaborations was the evaluation of current diagnostic methods against new emerging strains of NDV. The constantly developing network of laboratories in different countries around the world, collaborating on the control of the ND, resulted in the increasing number of samples obtained from both poultry (commercial and backyard) and wild birds. Thousands of collected specimens where tested by various serological, virological, and molecular methods. As a result, more than 350 NDV strains were isolated and most of them have been further studied and characterized. Most currently utilized rapid diagnostic methods based on rRT-PCR were effective in detecting ND viruses. The L-TET rRT-PCR designed to detect class I APMV-1 viruses (8) and multiplex rRT-PCR designed to detect both class I and class II APMV-1 viruses (9) have been successfully used for ND diagnostics. Furthermore, the modified fusion gene rRT-PCR (24) has been routinely applied in detecting of pigeon PMV-1 (25). However, the matrix gene rRT-PCR assay universally used and designed to detect all NDV class II strains (26), did not detect three isolates from Pakistan from 2006 to 2007 (14). A new matrix gene test with a modified probe that detected these isolates was developed (14). These viruses were later identified as members of a new genotype – XIII (7).

**Figure 1:**
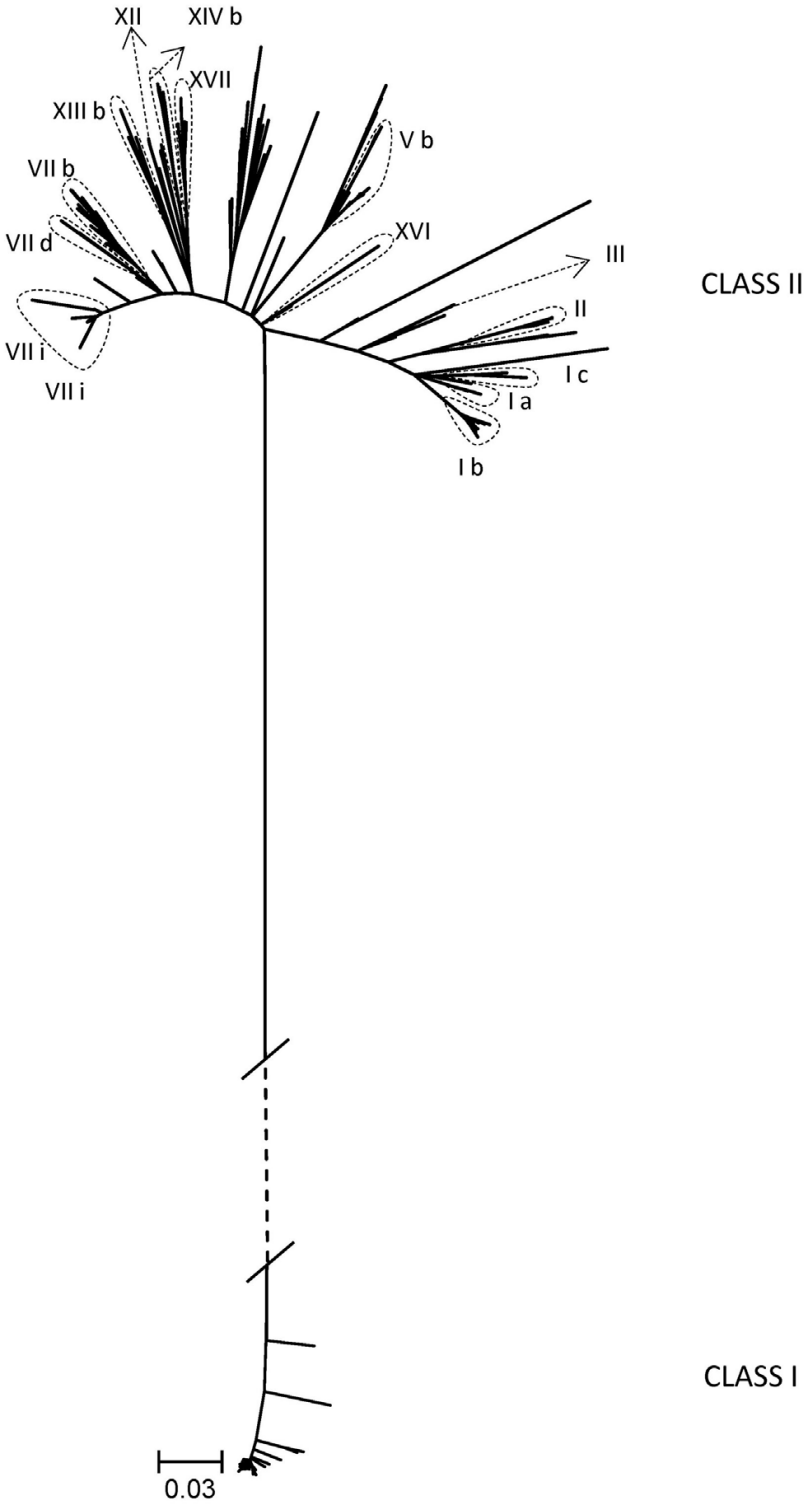
**Phylogenetic tree of complete coding sequences of the fusion gene of all viruses studied within the Biological Engagement Programs and additional collaborations (*n* **=** 183)**. Selected viruses from all known genotypes in class I and class II are also included in the tree (*n* = 94). The evolutionary history was inferred by using the Maximum Likelihood method based on General Time Reversible model as implemented in MEGA6 with 500 bootstrap replicates (29). The branch lengths represent the number of substitutions and are proportional to the differences between the isolates. The branch between class I and class II (dashed) is not drawn to scale. Evolutionary analyses were conducted in MEGA6 (30).

An additional goal of the BEP collaborations was the development of tools and knowledge to better understand the genetic evolution and molecular epidemiology of ND. Initial efforts were directed to obtain nucleotide sequences of different regions of the isolated strains’ genomes. In total, 222 partial fusion gene sequences from viruses from Indonesia, Kazakhstan, Kyrgyzstan, Mexico, Nigeria, Pakistan, Russia, and Ukraine and 18 matrix gene sequences from viruses from Pakistan and Mexico were obtained (18, 20, 22) (unpublished data). As the complete fusion gene sequence was later shown to provide more detailed and valuable information on ND epidemiology and NDV classification (7), this section of the NDV genome from 187 viruses was also sequenced. These viruses were collected from poultry and wild birds in Bulgaria, Indonesia, Israel, Japan, Malaysia, Mexico, Nigeria, Pakistan, Russia, U.S., and Ukraine and represented 17 different sub/genotypes within class I (*n* = 52) and class II (*n* = 135) (7, 10, 11, 13, 16, 23, 27) (unpublished data) (Table 2; Figure 1). The high diversity of viruses studied within the BEP and additional collaborations are represented in a phylogenetic tree including selected isolates from all known genotypes (Figure 1). As NDV is continuously evolving and leading to more diversity, new primers have been designed and tested to facilitate the sequencing of the complete fusion gene. The newly designed primers and also some previously published ones are presented in Tables 2 and 3, including the utilization scheme by sub/genotype. The availability of those primers should facilitate future detailed genetic characterization of NDV isolates.

**Table 2:** **Primer pairs used for sequencing the complete fusion gene of different Newcastle disease viruses from class I and class II**.

Primer pairs used for sequencing	CLASS II sub/genotype^1^																CLASS I
	I^a^	I^b^	I^c^	II	III	V^b^	VII	VII^b^	VII^d^	VII^i^	XII	XIII^b^	XIV^b^	XVI	XVII	XVII^b^	
MSF1/NDVR2	X		X	X	X	X	X				X	X					X

4331F/5090R^2^	X	X	X	X		X	X				X	X	X	X	X	X	X

4093F/4889R	X																

4707F/5518R	X		X														

4961F/5772R^2^	X	X	X	X		X							X	X	X		

4927F/5673R						X	X				X	X					

5358F/6178Ra	X		X														

5435F/6320R		X	X	X	X	X						X	X		X		

5491F/6341R					X		X				X	X					

5413F/6179R														X			

4317F/5078R				X													

4911F/5857R^3^				X													

5669F/6433R				X													

4008F/4994R								X	X	X							

4715F/5637R								X	X	X							

5410F/6332R						X		X	X	X		X					

4715F/6178Rb									X								

4963F/5832R						X											

5633F/6474R						X											

4927F/5641R					X	X	X					X				X	

5378F/6204R						X	X									X	

4504F/4859R										X							

4853F/5324R										X							

5258F/5609R										X							

5592F/5939R										X							

4353F/5129R										X		X					

5067F/5837R												X					

4089F/4895R										X							

4772F/5533R										X							

5412F/6311R										X							

4445F/5098R																	X

5969F/5748R																	X

5550F/6326R																	X

5550F^4^/6326R^5^																	X

5076F/5838R																	X

5643F/6354R																	X

*^1^The table represents primer pairs used in this manuscript to identify each genotype. Primer pairs not marked with an X may also identify isolates from other genotypes*.

*^2^Primers designation as published previously (8, 28)*.

*^3^The use of this set of primers did not result in PCR products for all tested genotype II isolates*.

*^4,5^Modified by Andrew Reeves (13)*.

*The sequences of all primers are presented in Table 3*.

**Table 3:** **Sequences of the primer pairs used for sequencing the complete fusion gene of different Newcastle disease viruses from class I and class II**.

Primer pair		Sequence (5**′**–3**′**)	Tm in °C	Reference
MSF1/NDVR2		GACCGCTGACCACGAGGTTA	55.9	(6)
		AGTCGGAGGATGTTGGCAGC	55.9	
4331F/5090R^1^		GAGGTTACCTCYACYAAGCTRGAGA	56–61	(8)
		TCATTAACAAAYTGCTGCATCTTCCCWAC	57.3– 58.7	
4093F/4889R		ATCTGTCGGGCTCAGCGACGTG	60.4	
		AGCGCCTATAAGGCGTCCCTG	58.3	
4707F/5518R		ACACCTCATCCCAGACAGGGTCG	60.6	
		CTATCACGGAACCGACCTGCGTC	60.6	
4961F/5772R^1^		GCTCTGATACARGCMAAMCAAA	49.2–54.8	(28)
		TGCGATATGATWCCCGGRG	51.1–53.2	
4927F/5673R		TCTTGGGGTTGCAACAGCGGCAC	60.6	
		GGCGTAGTGAGTGCACCTTCAG	58.6	
5358F/6178R^1^		CCGGCAACCCTATCCTGTACGAC	60.6	
		AGTGGCCCTCATCTGGTCCAGG	60.4	
5435F/6320R		AAYAATATGCGYGCCACCTACYTRG	54.4–61	
		ACCGTTCTACCCGTRTRTYGY	50.5–58.3	
5491F/6341R		TGCCTCAGCACTTGTCCCGAAAG	58.8	
		TCGATTGAAGGATGGCTCCTCTG	57.1	
5413F/6179R		TCTACCCTCAGTTGGGAACC	53.8	
		GTTGGCCCTCATCTGATCGAG	56.3	
4317F/5078R		CCGACCACGAGGTTACC	51.9	
		TGCATCTTCCCAACTGCCAC	53.8	
4911F/5857R		TTATTGGCGGTGTGGCTC	50.3	
		AGTTACATCGAATTCCCCACTG	53	
5669F/6433R		AAGACCGAAGGCGCACTTAC	53.8	
		TCTCTAACGCAACTTGGCT	48.9	
4008F/4994R		ATATCGGGCTTATGTCCACTG	52.4	
		CTTAAGCCGGAGGATGTTGGC	56.3	
4715F/5637R		TCTCAGACAGGGTCAATC	48	
		AAGCTGACGTATTGCCGCTCA	54.4	
5410F/6332R		GAATTTGCCCTCAGTCGGGA	53.8	(16)
		GTGGCTCCTCTGACCGTTCTA	56.3	
4715F/6178R^2^		TCTCAGACAGGGTCAATC	48	
		GTAGTGGCTCTCATCTGATCGAGG	59.1	
4963F/5832R		TGCAGCTCTGATACAAGCCAACC	57.1	
		AGCCTCAGGGTTATTCCGTCTAGGG	61	
5633F/6474R		TGTCTGAGCGGTAATACGTCAGCTTG	59.5	
		TGATCCGAAAAACCAAGCGCCATGTG	59.5	
4927F/5641R		TCTTGGGGTTGCAACAGCGGCAC	60.6	
		CATGCACGCTGACGTATTGCCG	58.6	
5378F/6204R		GACTCACAGACTCAACTCTTGG	54.8	
		CTCTCATCTGCGTTCATGCTC	54.4	
4504F/4859R		ACGGGTAGAAGATTCTGGATC	52.4	(16)
		CCTCCTGATGTGGACACAGCCCC	62.4	
4853F/5324R		ATACAGGGGGCTGTGTCCAC	55.9	(16)
		CTACCAATTAATGAGCTGAGTTG	51.7	
5258F/5609R		GCACTTTACAATCTAGCTGGT	50.5	(16)
		ATACCAGGGGACATAGG	47.1	
5592F/5939R		CAAGAATAGTAACATTCCCTATG	49.9	(16)
		ACATTCCCAAGCTCAGTTGA	49.7	
4353F/5129R		GGCACACCATTGCTAAATAC	49.7	(16)
		TATACAATCCAATTCTCGCGC	50.5	
5067F/5837R		CACAACTAGCAGTGGCAGT	51.1	(16)
		AGCCTCAGAGTTATCCCGTC	53.8	
4089F/4895R		ATCTATCTGTCGGGCTCAGTGAC	57.1	(16)
		GCCATTAACGGCACCTATAAAGCG	57.4	
4772F/5533R		GCGTGTGCAAAAGCCCCATTAG	56.7	(16)
		GAGGTGTCAAGTTCTTCTATCACG	55.7	
5412F/6311R		ATTTGCCCTCAGTCGGGAACC	56.3	(16)
		ACCCGTGTATCGTTCTTTGGTC	54.8	
4445F/5098R^2^		CACTAATCAAGTCTGATAATTGAACC	53.2	
		GTCRTTAACAAACTGCTGCATC	51.1–53	
5969F/5748R^1^,^2^,^3^		TTCTGCCCTCATACAAGCCAACC	57.1	
		GGGGGATCTGCGCACCTACACG	62.3	
5550F/6326R^2^		AGCTGGATACGTCATATTGCATAG	54	
		ACCGTTCTACCCGTRTATCGY	52.4–56.3	
5550F^2^,^3^	1 abr	AGCTGGATACATCGTATTGCATAG	54	Andrew Reeves, personal communication
	2 abr	AACTGGATACGTCATATTGCATAG	52.3	
6326R^2^,^3^	1 abr	ACTGTTCTACCCGTATATCTT	48.5	Andrew Reeves, personal communication
	2 abr	ACTGTTCTACCCGTATATATC	48.5	
	3 abr	ACYGTTCTACCCGTRTRTYGY	48.5–58.3	
5076F/5838R^2^		AGCTGGCTGTTGCCGTAGGT	55.9	
		CGCAAGGTGATCCCGTCGAGAG	60.4	
5643F/6354R^2^		TCGGTGGAAACACCTCAGCATGC	58.8	
		ACGCTCCTTGAATGGAGGCGAC	58.6	

*^1^Primers designation as published previously (8, 28)*.

*^2^Aligned to the complete genome of APMV-1/Goose/Alaska/415/91 (GenBank Acc.# AB524405) and designated accordingly*.

*^2,3^Modified by Andrew Reeves (13)*.

**T*_m_, melting temperature*.

*All primers (except class I^b^ primers) were aligned to the complete genome of APMV-1/Goose/China/ZJ1/2000 (GenBank Acc.# AF431744.3) and designated accordingly*.

## Conclusion

Newcastle disease represents an international problem and a significant threat to poultry industries worldwide. The existence of a large number of countries with endemic virulent ND circulating in poultry and wild birds, the capacity of viruses for rapid intercontinental spread, the ability of viruses to gradually change, and the potential use for bioterrorism, require the development of more internationally funded epidemiological and veterinary research programs. The BEP collaborative projects have effectively contributed to the understanding of the threat and to the development of better tools to identify and characterize this agent.

## Author Contributions

PM, KD, and CA defined the theme of the manuscript. All authors contributed on the data collection, writing of the manuscript and editing the manuscript. KD, PM, and DW-C created the tables. KD created the figure. DW-C, KD, and CA designed primers. All authors have seen and approved the manuscript.

## Conflict of Interest Statement

The authors declare that the research was conducted in the absence of any commercial or financial relationships that could be construed as a potential conflict of interest.

## Footnotes

^1^http://www.oie.int/wahis_2/public/wahid.php/Diseaseinformation/statuslist.
